# Service Evaluation Exploring the Use of Standardised Assessment Tools to Assess Non-Cognitive Symptoms of Dementia

**DOI:** 10.1192/bjo.2022.414

**Published:** 2022-06-20

**Authors:** Daniel Romeu, Amelia Taylor, Alexander Graham, Jane Chatterjee, Sonia Saraiva, Ben Underwood, Emma Wolverson, Gregor Russell, George Crowther

**Affiliations:** 1 Leeds and York Partnership NHS Foundation Trust, Leeds, United Kingdom; 2 Leeds Institute of Health Sciences, Leeds, United Kingdom; 3 South West Yorkshire NHS Foundation Trust, Wakefield, United Kingdom; 4 St Gemma's Hospice, Leeds, United Kingdom; 5 University of Cambridge, Cambridge, United Kingdom; 6 Humber Teaching NHS Foundation Trust, Hull, United Kingdom.; 7 University of Hull, Hull, United Kingdom; 8 Bradford District Care NHS Foundation Trust, Bradford, United Kingdom

## Abstract

**Aims:**

Pain, depression, anxiety, and psychosis are common non-cognitive symptoms of dementia. They are often underdiagnosed and can cause significant distress and carer strain. Numerous standardised assessment tools (SATs) exist and are recommended for the assessment of non-cognitive symptoms of dementia. Anecdotal evidence suggests that SATs are used rarely and inconsistently. This study aims to explore which SATs to detect non-cognitive symptoms of dementia are recommended in local guidelines and used in practice across different organisations. Secondary aims were to identify barriers and facilitators to using these tools.

**Methods:**

This service evaluation is cross-sectional in design. A questionnaire was developed and distributed to clinicians working with patients with advanced dementia in any setting, across four geographical locations (Leeds, Bradford, Hull, and Cambridge). Quantitative data were analysed descriptively, and qualitative data from free-text comments were interpreted using thematic analysis.

**Results:**

135 professionals from a range of backgrounds and clinical settings completed the survey. Respondents indicated that SATs for non-cognitive symptoms in dementia were rarely used or recommended. Respondents were unaware of the existence of most SATs listed. 80% respondents felt that SATs were a useful adjunct to a structured clinical assessment. The most recommended tool was the Abbey Pain Scale, with 41 respondents indicating its recommendation by their Trust. Perceived facilitators to using SATs include education and training, reliable IT systems and accessibility. Barriers include lack of time and training.

**Conclusion:**

Numerous SATs are available for use in dementia, but they are rarely recommended in local policy or used in practice. There appears to be a lack of consensus on which, if any, are superior diagnostic tools, and on how or when they should be applied.

